# Public Perception of Pharmacists in Poland

**DOI:** 10.3390/ijerph19052515

**Published:** 2022-02-22

**Authors:** Maja Andrzejewska, Urszula Religioni, Paweł Piątkiewicz, Agnieszka Barańska, Jolanta Herda, Aleksandra Czerw, Jerzy Krysiński, Piotr Merks

**Affiliations:** 1 Department of Pharmaceutical Technology, Faculty of Pharmacy, Collegium Medicum in Bydgoszcz, Nicolaus Copernicus University in Toruń, 85-089 Bydgoszcz, Poland; paszunmaja@gmail.com (M.A.); jerzy.krysinski@cm.umk.pl (J.K.); 2 School of Public Health, Centre of Postgraduate Medical Education of Warsaw, 01-813 Warsaw, Poland; 3 Collegium of Business Administration, Warsaw School of Economics, 02-554 Warsaw, Poland; 4 Department of Ophthalmology, Medical University of Warsaw, 02-097 Warsaw, Poland; piatkiewicz@op.pl; 5 Department of Medical Informatics and Statistics with E-Learning Lab, Medical University of Lublin, 20-090 Lublin, Poland; agnieszkabaranska@umlub.pl; 6 Department of Public Health, Medical University of Lublin, 20-090 Lublin, Poland; jolantaherda@umlub.pl; 7 Department of Health Economics and Medical Law, Medical University of Warsaw, 02-097 Warsaw, Poland; aleksandra.czerw@wum.edu.pl; 8 Department of Economic and System Analyses, National Institute of Public Health NIH—National Research Institute, 00-791 Warsaw, Poland; 9 Faculty of Medicine, Collegium Medicum, Cardinal Stefan Wyszyński University, 01-815 Warsaw, Poland; p.merks@uksw.edu.pl

**Keywords:** pharmacist, pharmaceutical care, patient, Poland

## Abstract

Background. Pharmacists constitute one of the largest groups of medical professionals and play a significant role in public health. Pharmaceutical care in community pharmacies is one of the key elements that impact the clinical outcomes of patients. The main objective of this study was to evaluate the public perception of pharmacists in Poland, as well as the knowledge of and willingness of Polish people to use pharmaceutical care services. Methods. This study was carried out in 2017 on 1435 people. The research tool was an anonymous online questionnaire. Results. Of the participants, 61% considered pharmacists to have a position of public trust, and 25% trusted pharmacists to a lesser extent than representatives of other medical professions. The participants stated that pharmacists were kind (74%) and helpful (69%). For 52% of the participants, pharmacists were fully competent to provide information on medications. Twenty-eight percent of the participants ask pharmacists for advice related to medicinal products. Poles’ knowledge on pharmaceutical care was low (44% of the respondents knew this notion). Sixty-six percent of the participants were willing to use pharmaceutical consultations (43% free of charge, and 23% for a nominal fee). Conclusions. Although the overall perception of patients towards pharmacists was positive in Poland, it is essential to educate patients on the possibilities of using pharmaceutical services, and to promote the role of pharmacists in healthcare systems.

## 1. Background

Pharmacists constitute one of the largest groups of medical professionals [1], providing such services as quality assessment of compounded, officinal, and ready-made medications, dispensing medical devices and medicinal products, giving information and advice, and providing pharmaceutical care [2,3]. Pursuant to Polish law, pharmaceutical care involves the process through which a pharmacist cooperates with a patient, the patient’s doctor, and, if necessary, representatives of other medical professions, supervising the appropriate course of pharmacotherapy in order to obtain the expected outcomes to improve the patient’s quality of life [4].

Over 32 thousand pharmacists are entered in the Polish Register of Pharmacists run by Regional Pharmaceutical Councils, of which more than 13 thousand are managers of community pharmacists [5]. Pharmacists most often work in community pharmacies, which involves everyday contact with patients and the resulting responsibility for patient health and life, as highlighted in Polish law [4]. This seems to be particularly significant, taking into account the number of prescription errors [6] and their health and economic consequences [7]. Pharmacist knowledge allows for optimizing treatment outcomes and preventing medication-related problems [8,9].

Liberalization of the pharmacy market in Poland has substantially increased the number of pharmacies and pharmacy chains [10], reducing the prestige of pharmacists and changing the perception of pharmacists having positions of public trust. For this reason, the objective of this study was to verify the knowledge of society in Poland about pharmacist education and professional responsibility as well as to evaluate the overall perception of this profession and patient satisfaction with pharmaceutical consultations and services. This study also aimed at testing patient knowledge on pharmaceutical care in Poland and their willingness to use such services as well as determining whether people in Poland trust pharmacists and use their assistance in choosing medications. It was essential to check the relationship between patient opinion on pharmacists in community pharmacies and in pharmacy chains, the dependencies between the perception of pharmacists and the use of their advice regarding medications, and the connection between patient chronic diseases and the evaluation of pharmacist competencies or willingness to use pharmaceutical care.

## 2. Methods

This study was carried out from March 2017 to May 2017. It was approved by the Bioethics Committee of the Ludwik Rydygier Medical College in Bydgoszcz (KB 144/2017). It was based on an anonymous author’s online questionnaire disseminated in social media (Facebook, Twitter). The survey was constructed on the basis of a literature review and consultation with two experts in the field of pharmacy and methodology. The questionnaire consisted of two parts and a section on personal data (sex, age, education, place of residence, use of pharmacy chains, chronic diseases). The criteria for including respondents in the study were a place of residence in Poland and age of over 18 years. The first part of the questionnaire concerned the evaluation of pharmacists and consisted of 14 questions related to the respondents’ knowledge on the education of pharmacists, pharmaceutical services, and the differences between pharmacy technicians and pharmacists. It also assessed the level of public trust in pharmacists. The second part aimed at evaluating pharmaceutical consultations and the level of patient satisfaction with pharmacy service. It consisted of 11 questions related to pharmaceutical care, advice, and information on medications provided by pharmacists. It also covered the factors influencing the patient’s choice of a pharmacy and patient awareness of cheaper medication equivalents.

Statistical analysis was performed with Statistica v.10. Dependencies between variables were tested with the use of a Chi-squared test, and the strength of association was measured using Cramer’s V. The level of statistical significance was taken at *p* < 0.05.

## 3. Results

### 3.1. Characteristics of the Sample

The study comprised 1435 people, of which 82% were women. The respondents below 24 years of age made up 41%, between 25 and 40 years—44%, and between 41 and 65 years—14%. The number of people aged 65 and over was small. Half of the respondents had higher education, 30% were in the course of their studies, 17% had secondary education, 2% had vocational education, and 1% had primary education. Fifty-three percent of the respondents lived in cities with over 100 thousand inhabitants, 22% lived in medium-sized cities, 10% in towns, and 15% in villages. 

Thirty-three percent of the patients reported chronic diseases, of which the most frequent were allergies, neurological disorders, cardiovascular diseases, and thyroid disorders.

Eighty-one percent of the respondents knew the difference between pharmacy chains and independent pharmacies. Thirty-seven percent declared visiting pharmacy chains, and 42% did not pay attention to the kind of pharmacy. Ten percent did not visit pharmacy chains, and 11% did not pay attention to the kind of pharmacy.

The characteristics of the study group are presented in Table 1.

### 3.2. Evaluation of the Pharmacists

Eighty-seven percent of the respondents knew that a pharmacist is a person with higher education. Eight percent were of the opinion that pharmacists attend post-secondary schools, 3% believed that they finished vocational schools, and 2% believed that they did not need any specialized education. The majority of the respondents (87%) were aware that pharmacist education lasts five and a half years and involves a half-year apprenticeship in a pharmacy. Eleven percent thought that pharmacists attended two-year post-secondary schooling, and 2% were of the opinion that the Matura exam is sufficient to become a pharmacist. Seventy-two percent declared knowledge of the difference between a chemist and a pharmacist. 

The vast majority of the respondents (84%) were aware that pharmacy technicians and pharmacists have different education and professional qualifications, 6% of the respondents thought that pharmacy technicians and pharmacists have different education yet the same professional qualifications, while 3% did not see any difference between the two professions, and 7% admitted that they did not pay attention to this issue.

Sixty-six percent of the respondents were aware of the different professional responsibility of pharmacists and technicians. For 33% of the respondents, name tags with academic titles worn by the pharmacy staff were important, while 22% who payed attention to them did not think they mattered. Thirty-two percent did not pay attention to the name tags, and 13% did not notice the name tags worn by pharmacy staff.

The appearance of the pharmacist was important for 57% of the respondents. Twenty-two percent of them paid attention to their age (a slightly lower percentage of the respondents preferred to be served by younger staff). For 86% of the respondents, the pharmacist’s gender did not matter. Twelver percent preferred to be served by men, and 2%—by women.

The most common noticed behavior was kindness (74%). According to 69% of the respondents, pharmacists were helpful and ready to give advice. A significantly lower percentage reported negative emotions in the pharmacists, which was indicated for both independent pharmacies and pharmacy chains (*p* > 0.05). Sixty-one percent of the respondents considered pharmacists to have a position of public trust, 25% trusted pharmacists, yet to a lesser extent than others. Fourteen percent did not treat pharmacists as though they held a position of public trust. These results were not differentiated by chronic diseases (*p* > 0.05).

More than half of the respondents (52%) were of the opinion that pharmacists are fully competent to provide information on medications. Forty-four percent thought that pharmacists are competent, yet they preferred to ask their doctor how they should take their medications. The remaining 4% stated that pharmacists were not the relevant people to provide information on medications, and these results were not differentiated by chronic diseases (*p* > 0.05).

Nearly all of the respondents (95%) knew that pharmacists can prepare medications from prescriptions, and 57% of them have used this form of service. Eleven percent were aware that in exceptional situations, pharmacists can dispense medicinal products on the basis of pharmaceutical prescriptions and have used this service. Forty-five percent did not have any knowledge in this respect.

The above-mentioned results are presented in Table 2.

### 3.3. Evaluation of Pharmaceutical Consultations and Patient Satisfaction in a Pharmacy

Every third respondent (33%) reported that they look for information on the use of medications in information leaflets. Twenty-eight percent of the respondents turned to pharmacists, and 25% to doctors. Every tenth patient always turned to pharmacists to ask about medication dosage and any adverse reactions, and 13% never asked pharmacists questions about medications. A significant dependence between having trust in the pharmacists and the choice of source of information about medications was observed in this study (*p* < 0.001; V = 0.30). Those respondents who indicated that pharmacists were competent usually looked for information on medications from pharmacists. Similarly, the respondents who considered pharmacists as having a position of public trust significantly more often consulted the choice of their medications with the pharmacists (*p* < 0.001; V = 0.22).

Every third patient reported that pharmacists always tell them how they should take their medications and inform of any adverse reactions, and 60% admitted that pharmacists give this information when they are asked to. Eleven percent of the respondents have never received this information from pharmacists.

The majority of the respondents (65%) asked the pharmacists for assistance when buying OTC medications and dietary supplements. Six percent of the respondents asked their doctors about these issues, and 29% admitted that they decide themselves.

The most important factor determining the choice of pharmacy were the prices of the medications (73%). For 55% of the respondents, a favorable location of the pharmacy was significant, for 35%—having a wide assortment, for 34%—professional pharmacist service, and for 31%—the opening hours. Being treated kindly by pharmacy staff (24%), efficient and fast service (16%), and appropriate pharmacy decor (1%) were less significant.

About 35% of the respondents were offered medication equivalents by pharmacists. Half of the respondents emphasized that they received this information after asking about this issue. Thirty-three percent of the patients were aware that not all medications can be substituted with cheaper generic medications and knew of these generics. Sixty percent of the respondents declared a lack of possibility of substituting some medications, and 7% did not know that there are medications that must not be substituted.

Forty-four percent of the respondents knew what pharmaceutical care entails. Forty-three percent were willing to use pharmaceutical consultations free of charge, 23% would use them if they were reimbursed or for a nominal fee, and 5% would pay for this service. The remaining 29% admitted that they would not use this service. This study does not indicate any correlation of the willingness to use pharmaceutical services between healthy people and those with chronic diseases (*p* < 0.05).

The results of the third part of the questionnaire are presented in Table 3.

## 4. Discussion

This study showed that patients in Poland had a positive attitude toward pharmacists and considered them to be helpful and kind. Patients obtained most information on medications from information leaflets and from their doctors. Pharmacists were relatively seldom asked about dosage and adverse reactions. This situation may result from the fact that pharmaceutical advice and pharmaceutical care are not promoted in Poland. Our study indicates that the majority of the respondents had little knowledge on pharmaceutical care. A number of the respondents would take part in a pharmacist consultation, provided that this service was free of charge or for a nominal fee. 

Pharmacists fulfil a very important role in healthcare [11,12], supporting doctors and patients in optimal and reasonable pharmacotherapy and serving as counsellors in pharmacotherapy [13,14]. In recent years, pharmacists have been engaging in wide promotion of healthy lifestyles, supporting patients in ceasing smoking and fighting obesity [13,15]. The pharmaceutical care practiced in many other countries worldwide [16] is slowly but surely being implemented in Poland. As part of such pharmaceutical care, pharmacists conduct medication reviews, talk to patients about their health and wellbeing, check their blood pressure, and take part in a variety of health-promotion programs.

In this way, the role and perception of pharmacists in healthcare is changing, from one of just dispensing medications to one of a person participating in the patient treatment process. These activities are particularly important considering the frequency of self-medicating among patients. A pharmacist is often the first person a patient turns to with a health problem, as a result of impeded contact with a doctor. The pharmacist is also the last person a patient is in contact with before deciding to take a given medication. The role of pharmacists is of special significance during the COVID-19 pandemic, where access to many other healthcare facilities is hindered [17,18]. 

This situation is of particular importance taking into account the scale of self-treatment among patients and their relatively low awareness of medications [19]. Studies carried out in Poland indicate that patients more and more often report adverse reactions and doubts regarding medications to pharmacists [20]. Pharmacist advice seems essential in the face of common advertisements of OTC medications and dietary supplements in mass media [21]. Studies prove that half of Poles are directed by advertisements when buying OTC medications and dietary supplements [22]. 

Our study shows the overall positive perception of pharmacists by the respondents. These results coincide with studies by Pronk et al. carried out among pharmacy patients in the Netherlands, where the respondents stated that pharmacists were helpful and friendly, and that they were satisfied with the services they provided [23]. In another study by Zimmerman, the vast majority of patients assessed pharmacist advice as exhaustive and comprehensible [24]. Similar results have been published in other countries, e.g., in Canada [25]. Comparing the obtained results with those from countries located in the Eastern and Europe, it was found that the perception of pharmacists and satisfaction with pharmaceutical care were at a similar level. For example, in Slovakia, 82.0% of patients indicated they were satisfied with the care provided by their pharmacists, and more than 73% of patients considered the pharmacist an expert in the field of medicines [26]. On the other hand, the research conducted in Slovenia proves the high quality of pharmaceutical care in community pharmacies [27].

Despite the fact that in our own study the majority of the respondents (61%) trusted pharmacists to the same extent as others in public trust positions, one fourth expressed that they trusted pharmacists less than representatives of other medical professions. Similarly, most of the respondents preferred advice from a doctor when choosing a treatment, as confirmed by Szalonka et al. [28]. The results of our own study substantially deviate from those of Perepelkin in Canada, where the vast majority of the respondents agreed with the statement that pharmacists are the same healthcare professionals as doctors and nurses, and that pharmacists have more extensive knowledge on pharmacotherapy than doctors. In the event when a pharmacist and a doctor have different opinions, the respondents preferred to follow the pharmacist’s advice [29]. A lot of respondents in Canada proposed further development of pharmaceutical services [30]. 

So far, only several studies have been conducted in Poland on the perception of medical professions in Poland. For example, the study by Łosiewicz and Ryłko-Kurpiewska shows that the level of trust of the Polish society towards doctors remains relatively low [31]. Slightly different results are indicated by Krajewska-Kułak et al., in whose studies 25.3% of Polish patients did not doubt in proper medical care. At the same time, however, 90% of respondents agreed with the statement “I trust my doctor very much, therefore I always follow his advice” (the study concerned gynecologists) [32]. Similar results were obtained by Marcinowicz et al., examining the patients’ trust in general practitioners [33].

Our own study indicates that professional patient service in pharmacies is not an important determinant when choosing the place to buy medications, as most patients are directed by price, which coincides with Szalonka et al., where location and price were the most important factors for patients, and professional advice and friendly atmosphere mattered less [28]. Piecuch et al. [34] also show the price as the major criterium of selection of medicinal products. Opposite results can be observed in studies carried out abroad. In Perepelkin’s study in Canada, patients indicated such factors as pharmacist experience, conversation with a pharmacist on a prescription, and the treatment by pharmacy staff [29]. In a study by Khan et al. carried out among patients in Pakistan, price and advice given by trained pharmacists were the most important factors [35]. Interesting results were presented by Merks et al., where factors determining the choice of pharmacy among patients in Poland and Great Britain were compared. British patients more often chose criteria such as good advice from pharmacists and the possibility of discussing health problems with pharmacists. Prices of medications were much less important than in the case of the respondents in Poland [36]. 

Our own study shows that 56% of the respondents did not know what pharmaceutical care was, which may result from the fact that pharmaceutical care in Poland is not legally regulated or funded by a third-party payer. Other results were presented by Cerbin-Koczorowska et al. among inhabitants of three cities in Poland [37]. The majority of the respondents expressed a need for the implementation of pharmaceutical care in pharmacies in Poland. The vast majority of the respondents were not willing to pay for this and expected that this would be paid by the insurer. Other studies conducted in Poland show patient openness to health education by pharmacists that would involve issues related to medications and lifestyle [38]. Similar results are observed in another study carried out in Poland, where the vast majority of the respondents (85%) agreed to the implementation of pharmaceutical care to pharmacies in Poland, and nearly half of the respondents expressed their readiness to pay for pharmaceutical care services, though the declared amounts were nominal and oscillated from a few to several dozen zloty [39]. Similar results were obtained in a study carried out in Serbia, where 38% of the respondents expressed their willingness to pay for pharmaceutical care, and their suggested fees were from one to two dollars [40]. A different point of view is presented in studies carried out in Great Britain by Rodgers et al. Despite the fact that for the majority of the respondents filling prescriptions and buying medications are the main reasons of their visit to a pharmacy, over two thirds of the patients admitted that they also went to pharmacies for advice on medications and health problems. More than 90% of the respondents expressed their willingness to use pharmacist services in the future. They expected that consultations with pharmacists would expand their knowledge on their medications, help in understanding the mechanisms of action of their medications, and advise on how they should take their medications. Those respondents who had already used pharmacist services believed that their perception of pharmacists as a medical profession has changed, and their awareness of pharmacist knowledge increased [41], which may result from the extended services offered by pharmacists in Great Britain. Similar results are presented in studies in Canada [42]—as pharmacists’ responsibility and the scope of their duties were increasing, the respondents perceived them as more and more professional. Literature indicates that perceiving pharmacists as healthcare professionals substantially increases patient adherence and brings better treatment outcomes [43,44].

## 5. Limitations

Our study, despite our best efforts, has several limitations. First of all, the group of respondents was mainly women. Moreover, only a few were over 65 years old. These factors may influence the obtained results. 

Additionally, due to a lack of a standardized questionnaire evaluating attitudes to pharmacists, we decided to develop our own survey. Despite the fact that the project of our survey was consulted with healthcare representatives, we are aware that it may contain some imperfections. 

Considering the low number of studies on the public perception of pharmacists in Poland, we based our discussion on available yet relatively old studies. Our objective is to emphasize the necessity for further studies in this respect in order to fully understand attitudes toward pharmacists, and also determine the expectations related to the supply of pharmaceutical services. The organization of services that can improve patient treatment outcomes to the largest extent possible will only be attainable when we fully understand patient needs and expectations.

## 6. Conclusions

The conducted study indicated that people in Poland had great knowledge on the role of pharmacists in healthcare. Poles perceived pharmacists as an important and necessary professional group and considered them to be kind and professional. The majority of the respondents expressed their large trust in pharmacists. Nevertheless, information leaflets remained the main source of information on medications. Therefore, it is essential to educate patients in available pharmaceutical services and disseminate information on the benefits of pharmaceutical care for patients and healthcare systems.

Given the low number of articles on this topic, this work extends the literature in this area and provides valuable implications for research and practice of public health/pharmaceutical services in Poland.

## Figures and Tables

**Table 1:** Characteristics of the participants.

Variable	N (1435)	%
Sex		
F	1177	82.02
M	258	17.98
Age		
18–24	594	41.39
25–40	632	44.04
41–65	199	13.87
66+	10	0.70
Education		
Primary	13	0.91
Secondary	239	16.66
In the course of your studies	426	29.69
Higher	721	50.24
Vocational	36	2.40
Place of residence		
Rural	214	14.91
Sub-urban	149	10.38
Semi-urban	318	22.16
Urban	754	52.54
Do you suffer from chronic diseases?		
No	963	67.11
Yes	472	32.89
Do you know the difference between a pharmacy chain and an independent pharmacy?		
No	269	18.75
Yes	1166	81.25
Have you bought medications in a pharmacy chain (a pharmacy which groups a minimum of five outlets under one banner)?		
I don’t pay attention to which pharmacy I visit	600	41.81
No	147	10.24
I don’t know what kind of pharmacy I visit	154	10.73
Yes	534	37.21

**Table 2:** Evaluation of pharmacists.

Variable	N (1435)	%
What education does a pharmacist working in a pharmacy need to have?		
A pharmacist is a seller and doesn’t have to have any education	23	1.60
Post-secondary	111	7.74
Secondary vocational	46	3.21
Higher	1255	87.46
How long does pharmacist education last?		
They need to graduate from a 2-year post-secondary school	154	10.73
They need to complete a 5.5-year study at a university (including a half-year apprenticeship in a pharmacy)	1257	87.20
The Matura exam is sufficient	24	1.67
Do you know the difference between a chemist and a pharmacist?		
No	402	28.01
Yes	1033	71.99
Do you notice the difference between a pharmacy technician and a pharmacist?		
It doesn’t matter to me	103	7.18
No, I think that pharmacy technicians and pharmacists have the same education and professional qualifications	38	2.65
Yes, pharmacy technicians and pharmacists have different education, but the same professional qualifications	85	5.92
Yes, pharmacy technicians and pharmacists have different education and professional qualifications	1209	84.25
Do you think that pharmacy technicians and pharmacists bear the same professional responsibility?		
No	953	66.41
Yes	482	33.59
Do you pay attention to the name tags with academic titles worn by pharmacy staff?		
I haven’t noticed any name tags worn by pharmacy staff	192	13.38
No, I don’t pay attention to this	456	31.78
Yes, but it doesn’t matter to me	316	22.02
Yes, it matters to me	471	32.82
Do you pay attention to pharmacist appearance?		
No, it doesn’t matter to me	622	43.34
Yes, this is very important for me	813	56.66
Do you pay attention to pharmacist age?		
Yes, I prefer to be served by young staff	187	13.03
Yes, I prefer to be served by older staff	124	8.64
Pharmacist age doesn’t matter to me	1124	78.33
Do you pay attention to pharmacist gender?		
Pharmacist gender doesn’t matter to me	1228	85.57
Yes, I prefer to be served by women	171	11.92
Yes, I prefer to be served by men	36	2.41
Which pharmacist attitude do you most often observe when you visit a pharmacy?		
Kind, friendly, nice	1063	74.08
Offering advice and help	986	68.71
Listening carefully to patients	516	35.96
Professional, with extensive knowledge	569	39.65
Indifferent, bored	219	15.26
Not very kind or communicative	93	6.48
Insistent, wanting to sell as many medications as possible	84	5.85
I don’t pay attention to this	63	4.39
Do you consider pharmacists hold positions of public trust?		
Yes, I treat pharmacists on par with other positions of public trust (e.g., doctors, nurses)	883	61.43
Yes, but I trust pharmacists less than other positions of public trust	356	24.81
I don’t consider pharmacists hold positions of public trust	196	13.66
Do you consider pharmacists to be fully competent to provide information on medications?		
Pharmacists are competent, but I prefer to ask my doctor	631	43.97
I don’t consider pharmacists to be sufficiently competent to provide this information	56	3.90
Yes, I first turn to pharmacists when I have such questions	748	52.13
Do you know that pharmacists can prepare medications on the basis of prescriptions?		
I’ve never heard of this possibility	67	4.67
Yes, I’ve already taken medications prepared by pharmacists	827	57.63
Yes, I’ve heard of this possibility, but I’ve never taken medications prepared by pharmacists	541	37.70
Do you know that in exceptional situations pharmacists can dispense medications on the basis of pharmaceutical prescriptions?		
I didn’t know	642	44.74
I’ve heard of this, but I’ve never used this service	633	44.11
Yes, I know, and I’ve used this service	160	11.15

**Table 3:** Evaluation of pharmaceutical consultations and the level of patient satisfaction with pharmacy service.

Variable	N (1435)	%
Who do you turn to when you have questions and doubts regarding medications?		
To a pharmacist	395	27.53
To a doctor	361	25.16
To a nurse	9	0.63
To a pharmacy technician	18	1.25
I look for information online	174	12.13
I look for information in information leaflets	478	33.31
How often do you turn to pharmacists when you have questions regarding your medication dosage and any adverse reactions?		
I sometimes ask about these issues	614	42.79
I never ask questions about medications	183	12.75
I rather don’t ask questions about these issues	509	35.47
I always ask about these issues	129	8.99
Do pharmacists tell you how you should take your medication and inform you about any adverse reactions?		
I’ve never been given this information, even when I asked about it	158	11.01
Yes, but only if I ask about this	867	60.42
Yes, they always give me this information	410	28.57
Do you ask pharmacists for help when you buy OTC medications and dietary supplements?		
No, I always choose these products by myself	423	29.48
No, I prefer to ask my doctor	85	5.92
Yes, I often ask pharmacists for help	927	64.60
Is your choice of medications or dietary supplements influenced by pharmacist advice and opinion?		
No, because I don’t get exhaustive information	150	10.45
No, because I don’t need any advice in self-treatment	259	18.05
Yes, because they are professionals with extensive knowledge	1026	71.60
Please select the most important factor determining your choice of pharmacy		
Prices of medications	1046	72.89
Favourable location	795	55.40
Professional advice	490	34.15
Opening hours	452	31.50
Fast and efficient service	227	15.82
A wide assortment, without the necessity of returning for missing medications	505	35.19
Pharmacy decor	18	1.25
Being treated kindly by pharmacy staff	349	24.32
Do long queues in pharmacies make you irritated?		
No, I understand that pharmacists need time to dispense medications and give advice	812	56.59
Yes, this makes me irritated, because fast service in pharmacies is important for me	623	43.41
Do pharmacists offer you cheaper alternatives?		
Rather not	216	15.05
Yes, if I ask about this	676	47.11
Yes, always	507	35.33
Definitely not	36	2.51
Do you know that not all medications can be substituted by their cheaper alternatives?		
I didn’t know that some medications cannot be substituted	106	7.39
Yes, but I don’t know which medications can be substituted	858	59.79
Yes, I know which can be substituted, and which cannot	471	32.82
Do you know what pharmaceutical care is?		
No	804	56.03
Yes	631	43.97
Would you consult your health and medications with pharmacists?		
No, I wouldn’t	415	28.92
Yes, but only if this is free of charge	610	42.51
Yes, if this is partially reimbursed, and the fee is nominal	335	23.34
Yes, even if this is fully paid by patients	75	5.23

## Data Availability

All data are available from the corresponding author.
